# New Fast ApEn and SampEn Entropy Algorithms Implementation and Their Application to Supercomputer Power Consumption

**DOI:** 10.3390/e22080863

**Published:** 2020-08-05

**Authors:** Jiří Tomčala

**Affiliations:** IT4Innovations, VSB—Technical University of Ostrava, 17.listopadu 2172/15, 70833 Ostrava-Poruba, Czech Republic; jiri.tomcala@vsb.cz

**Keywords:** entropy, measure of complexity, approximate entropy, sample entropy, fast approximate entropy, fast sample entropy, benchmarking, software comparison, supercomputer power consumption

## Abstract

Approximate Entropy and especially Sample Entropy are recently frequently used algorithms for calculating the measure of complexity of a time series. A lesser known fact is that there are also accelerated modifications of these two algorithms, namely Fast Approximate Entropy and Fast Sample Entropy. All these algorithms are effectively implemented in the R software package TSEntropies. This paper contains not only an explanation of all these algorithms, but also the principle of their acceleration. Furthermore, the paper contains a description of the functions of this software package and their parameters, as well as simple examples of using this software package to calculate these measures of complexity of an artificial time series and the time series of a complex real-world system represented by the course of supercomputer infrastructure power consumption. These time series were also used to test the speed of this package and to compare its speed with another R package pracma. The results show that TSEntropies is up to 100 times faster than pracma and another important result is that the computational times of the new Fast Approximate Entropy and Fast Sample Entropy algorithms are up to 500 times lower than the computational times of their original versions. At the very end of this paper, the possible use of this software package TSEntropies is proposed.

## 1. Introduction

Nowadays, it is necessary in many scientific fields to find out whether a certain time series is chaotic and what is the degree of its chaotic behavior. There are several tests to detect chaotic dynamics of time series such as a 0–1 test for chaos [1] or Shilnikov chaos condition [2]. However, these tests can only be used to distinguish between regular and chaotic dynamics. However, if it is necessary to determine the level of determinism in the analyzed time series, it would be most appropriate to calculate its *entropy*.

In general, the entropy of a system determines the degree of its disorder [3]. This state variable is the higher the more disordered the system is. This level of disorder then proportionally affects the degree of predictability of such a system. The characteristics of this system are reflected in the time series created by this system, so with some simplification it can be argued that the entropy of this time series would be a measure of the unpredictability of this system and also a measure of its *complexity*.

In recent years, the often used algorithms for calculation of a measure of time series complexity are Approximate Entropy (ApEn) [4] and Sample Entropy (SampEn) [5]. At this point, however, it is important to say that these algorithms do not calculate entropy in a mathematical sense, but essentially determine the degree of complexity of the analyzed time series in various ways.

The Approximate Entropy was originally developed to analyze medical data, such as heart rate, and later its application was extended in finance, psychology, etc. It is defined as follows:

ApEn(x,m,r)=

(1) $$=\frac{1}{N-m+1}\left[\sum_{i=1}^{N-m+1}\log\left(\frac{|j_{i}|}{N-m+1}\right)\right]-\frac{1}{N-m}\left[\sum_{i=1}^{N-m}\log\left(\frac{|k_{i}|}{N-m}\right)\right],$$

where

$$j_{i}=\{\xi |∥y_{i}-y_{\xi }∥\leq r\wedge \xi \in \langle 1,N-m+1\rangle \},\;k_{i}=\{\xi |∥z_{i}-z_{\xi }∥\leq r\wedge \xi \in \langle 1,N-m\rangle \},\;y_{i}=\left[x_{i},x_{i+1},\ldots ,x_{i+m-1}\right],\;z_{i}=\left[x_{i},x_{i+1},\ldots ,x_{i+m}\right],\;N=\left|x\right|.$$

This Equation (1) looks for similar sub-sequences $y_{i}$ resp. $z_{i}$ of lengths *m* resp. m+1. If it is assumed that the evaluation of $∥y_{i}-y_{\xi }∥\leq r$ is one elementary operation, then N−m+1 operations are required to calculate each $|j_{i}|$. The total number of operations would then be $2N^{2}+N\left(6-4m\right)+2m^{2}-6m+7$, so the time complexity of the Approximate Entropy is $O(N^{2})$.

Another algorithm used for time series complexity analysis is the Sample Entropy, which is slightly simpler than the Approximate Entropy algorithm. Although it requires fewer operations to calculate, it will be shown below that it has the same time complexity. This algorithm was proposed in 2000 by Richman and Moorman to assess the complexity of physiological time series.

The definition of Sample Entropy is:

(2) $$SampEn\left(x,m,r\right)=\log\left(\frac{\sum_{i=1}^{N-m+1}\left|b_{i}\right|}{\sum_{i=1}^{N-m}\left|a_{i}\right|}\right),$$

where

$$b_{i}=\{\xi |∥y_{i}-y_{\xi }∥\leq r\wedge \xi \in \langle 1,N-m+1\rangle \backslash i\},\;a_{i}=\{\xi |∥z_{i}-z_{\xi }∥\leq r\wedge \xi \in \langle 1,N-m\rangle \backslash i\},\;y_{i}=\left[x_{i},x_{i+1},\ldots ,x_{i+m-1}\right],\;z_{i}=\left[x_{i},x_{i+1},\ldots ,x_{i+m}\right],\;N=\left|x\right|.$$

Note that sets $b_{i}$ and $a_{i}$ are different from sets $j_{i}$ and $k_{i}$ of Approximate Entropy. They do not contain index *i*, so calculating $|b_{i}|$ resp. $|a_{i}|$ takes only N−m resp. N−m−1 operations. However, it also brings with it the possibility that the sum of all $|a_{i}|$ can be zero. The total number of operations is then $2N^{2}+N\left(2-4m\right)+2m^{2}-2m+1$ and the time complexity is $O(N^{2})$ again.

## 2. New Fast Algorithms

Quadratic time complexity of the Approximate and Sample Entropy algorithms was the main reason why their accelerated modifications *Fast Approximate Entropy* and *Fast Sample Entropy* have been proposed in [6]. The original Approximate Entropy algorithm looks for mutually similar sub-sequences by comparing all possible sub-sequences with each other. This method is apparently an extremely time-consuming and also the same pair of sub-sequences is unnecessarily compared twice. The acceleration of the modified Fast Approximate Entropy algorithm is that, once it finds two similar sub-sequences, all other sub-sequences in the same neighborhood will be marked as already included in some neighborhood. An important fact is that these sub-sequences are no longer taken into account when searching for other similar sub-sequences, thereby speeding up these further searches.

The formula of the Fast Approximate Entropy then reads as follows:

FastApEn(x,m,r)=

(3) $$=\frac{1}{N_{m}}\left[\sum_{i=1}^{N_{m}}\log\left(\frac{|s_{i,m}|}{N_{m}}\right)\right]-\frac{1}{N_{m+1}}\left[\sum_{i=1}^{N_{m+1}}\log\left(\frac{|s_{i,m+1}|}{N_{m+1}}\right)\right],$$

where

$$s_{i,m}=\{\xi |(∥y_{i}-y_{\xi }∥\leq r)\wedge \left(\xi \notin s_{j,m},j<i\right)\},\;y_{i}=\left[x_{i},x_{i+1},\ldots ,x_{i+m-1}\right],$$

$s_{i,m}$ is a set of sub-sequences of length *m* belonging to the *i*-th neighborhood, and $N_{m}$ is number of these neighborhoods.

The same principle is used in the modified Fast Sample Entropy algorithm:

(4) $$FastSampEn\left(x,m,r\right)=\log\left(\frac{\sum_{i=1}^{N_{m}}\left|s_{i,m}\right|}{\sum_{i=1}^{N_{m+1}}\left|s_{i,m+1}\right|}\right),$$

where

$$s_{i,m}=\{\xi |(∥y_{i}-y_{\xi }∥\leq r,\xi \neq i)\wedge \left(\xi \notin s_{j,m},j<i\right)\},\;y_{i}=\left[x_{i},x_{i+1},\ldots ,x_{i+m-1}\right],$$

$s_{i,m}$ is a set of sub-sequences of length *m* belonging to the *i*-th neighborhood, and $N_{m}$ is number of these neighborhoods.

In this way, the search time for similar sub-sequences and thus the total calculation times can be significantly reduced. The nature of this modification implies that the number of operations is different for different time series of the same length, but the number of operations for the best case and for the worst can be determined. For the best case, the number of operations required to calculate both accelerated algorithms is equal to 2N−2m+7. In the worst case, the number of operations required to compute Fast Approximate Entropy is equal to $N^{2}+N\left(5-2m\right)+m^{2}-5m+7$ and to compute Fast Sample Entropy equals $N^{2}+N\left(2-2m\right)+m^{2}-2m+1$.

Thus, it can be seen from the above that the time complexity of both accelerated algorithms ranges from O(N) to $O(N^{2})$. An interesting fact is that the above-mentioned best case corresponds to a time series with a low degree of disorder, which also implies a low FastApEn or FastSampEn value of analyzed time series. On the contrary, the worst case corresponds to a time series with a high degree of disorder and hence a high FastApEn or FastSampEn value. The amount of computational operations and the time complexity of these accelerated algorithms is thus interestingly dependent on the value they calculate.

## 3. Supercomputer Power Consumption Time Series

For the purposes of this article, access to the time series representing the electrical consumption of its infrastructures [7] was obtained. The power consumption of this supercomputer depends on many facts that may depend nonlinearly on each other, and the number of such facts is so large that it is intractable for modeling, so it can be said that this supercomputer infrastructure is so called *complex system* in terms of its power consumption. Thus, the recorded time series of electrical consumption is a time series with complex dynamics, or simply a *complex time series*.

For this reason, this time series is a good representative of real-world time series for testing and comparing the performance of complexity degree analyzers. The results of these tests and performance comparison are shown in Section 4.

At this point, it should be mentioned why an analysis of a complexity degree of this time series of power consumption is useful. This analysis is usually used in medicine where the course of an electrocardiogram (ECG) or electroencephalogram (EEG) is analyzed. For example, based on EEG complexity degree, the beginning, end, and the type of epileptic seizure can be identified.

However, as shown in [8], the time series complexity can also be an indicator of its predictability in forecasting its future course. A high degree of the complexity in such a case suggests that any prediction at a given point in the time series is very likely to be far more erroneous than a prediction at another point in the analyzed time series where the complexity degree is lower. One could even say that, from a certain degree of time series complexity, any forecast is worthless and can easily be replaced by a simple arithmetic mean.

By analyzing the complexity of this time series of power consumption, it is possible to determine in advance whether the prediction of its development can be credible. This is very important if the forecast indicates the possibility of overloading the power system.

Accelerated versions of entropy algorithms such as Fast Approximate Entropy and Fast Sample Entropy from the software package **TSEntropies** [9] may be helpful in really quickly determining the plausibility of future time series predictions. See Appendix A for installation instructions.

**Figure 1:**
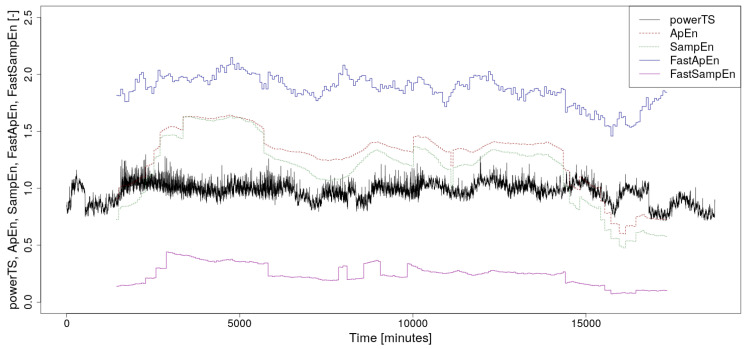
The normalized supercomputer power consumption time series, which is the subject of the analysis, along with calculated values of Approximate Entropy (ApEn), Sample Entropy (SampEn), Fast Approximate Entropy (FastApEn), and Fast Sample Entropy (FastSampEn). A floating window with a width of 2880 min was used for the calculation.

The calculated waveforms of ApEn, SampEn, FastApEn, and FastSampEn values together with the course of the analyzed power consumption time series are shown in the graph in Figure 1. As can be seen from this image, the values of the new accelerated algorithms are at different levels than their original versions. This fact is discussed in detail in [6], where it is also shown that their ability to detect an increase or decrease in complexity of the time series is roughly the same as in their original versions. A more detailed analysis of this phenomenon goes beyond the scope of this article.

Here is an example of a simple source code, which can be used to calculate the course of the Sample Entropy of power consumption, the measured values of which are stored in the time series *powerTS*. The floating calculation window in this case is 2880 in length:

library(TSEntropies)

SampEn_powerTS <- numeric()

for (i in 1:7120) {

SampEn_powerTS[i] <- SampEn(powerTS[i:(i+2880)])

}

plot(powerTS[1:10000], type="l")

lines(x = 1440 + (1:length(SampEn_powerTS)), y = SampEn_powerTS, type = "l",

lty = 3, col = "darkgreen")

# x = 1440 + ... is the offset to the middle of the calculation window

This chunk of code assumes that the power consumption time series *powerTS* is already normalized, which should be done in advance. The displayed course of the calculated SampEn value is shifted by half a calculation window so that its values in the graph are located in the middle of the area from which they were calculated.

## 4. Benchmarks and Comparison

Another software program that can calculate both Approximate Entropy and Sample Entropy is the R package **pracma** [10]. Therefore, this R package was chosen for comparison with the **TSEntropies** package. The **pracma** package is a versatile software that allows one to calculate many different practical features. It provides a large number of functions from numerical analysis and linear algebra, numerical optimization, differential equations, time series, plus some well-known special mathematical functions.

Three types of time series were used in the comparative tests. Firstly, the real-world time series *powerTS* represented by the power consumption of the supercomputer infrastructure. Furthermore, an artificial time series *sinTS* whose course is determined by the sine function. This time series is assumed to be a low degree of complexity. It will be interesting to observe the difference in computational times compared to the last type of time series *rnormTS* with a high degree of complexity, which is a random signal with normal probability distribution.

From the point of view of the focus of this paper, the most important are calculations of ApEn, SampEn, FastApEn, and FastSampEn values of the supercomputer power consumption time series *powerTS* from the real world and therefore the attention will be given especially on them. The detailed results of calculations of artificially created time series as *sinTS* and *rnormTS* have already been published in [6].

**Figure 2:**
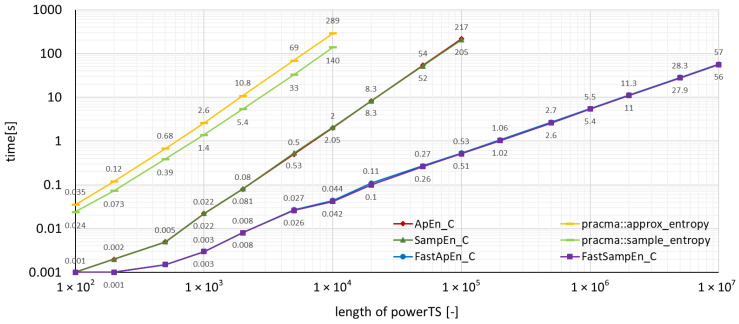
Comparison of the time needed to run various types of algorithms using the TSEntropies package functions implemented in C and the pracma package functions. The analyzed time series is the power consumption of the supercomputer.

**Figure 3:**
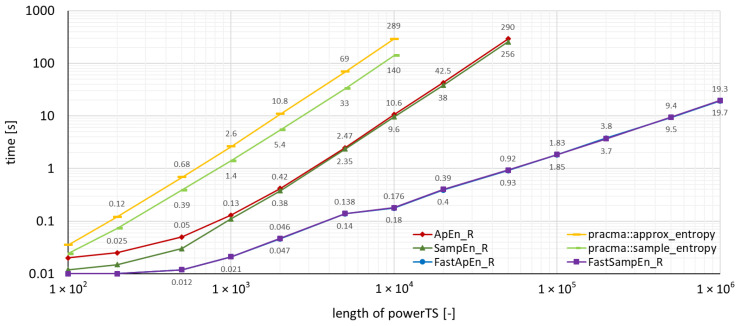
Comparison of the time needed to run various types of algorithms using the TSEntropies package functions implemented in R and the pracma package functions. The analyzed time series is the power consumption of the supercomputer.

The measured run-times for time series *powerTS* are depicted in the graphs in Figure 2, where the package functions implemented in C were used, and, in Figure 3, where the package functions implemented in R were used. The run-times achieved by the package **pracma** can also be found in these two figures, which allows an immediate graphical comparison of the performance with the **TSEntropies** package.

As mentioned in Section 2, the number of computational operations and hence the computational time required for new modified algorithms depends on the degree of disorder in the analyzed time series. This is one of the reasons why all three types of these time series were used to test the performance of this software. Since the lowest level of disorder is assumed for the *sinTS*, it is expected that its run-times will also be the lowest.

**Figure 4:**
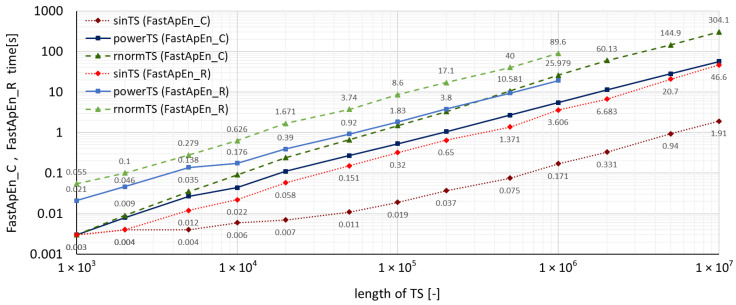
Comparison of Fast Approximate Entropy computation times for time series with various levels of disorder.

**Figure 5:**
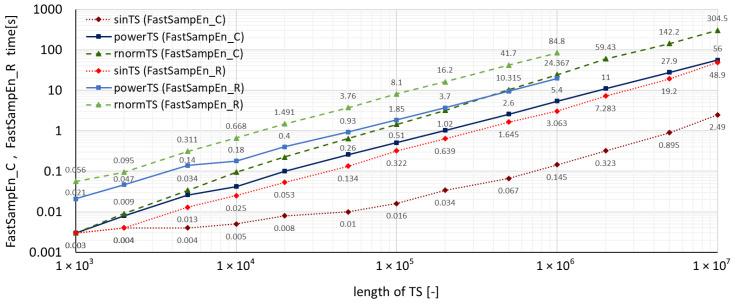
Comparison of Fast Sample Entropy computation times for time series with various levels of disorder.

A summary of measured run-times required by both accelerated algorithms for all three types of time series using the **TSEntropies** package is shown in Figure 4, which presents the resulting run-times of the Fast Approximate Entropy algorithm, and Figure 5, which presents the resulting run times of the Fast Sample Entropy algorithm. These calculations were performed for various lengths of time series from $10^{3}$ to $10^{7}$ samples.

During the calculations, the default values of the parameters were set for the executed functions. Their settings can be seen in Appendix B.

The calculations were performed on a computer with an Intel Core i5-6200U CPU @ 2.30 GHz processor, 8 GB RAM SO-DIMM DDR3 1600 MHz and SSD LiteOn L8H-256V2G-HP. The operating system installed was Ubuntu 18.04.4 (64-bit).

This testing could, of course, be done on one of the supercomputer clusters (Salomon, Anselm, or Barbora) and surely shorter computational times would be achieved, but there was an effort to show that this R package **TSEntropies** and also new algorithms can handle any time series even on a regular computer.

## 5. Conclusions and Future Work

As can be seen from the graphs of run-times in Figure 2, Figure 3, Figure 4 and Figure 5, the **TSEntropies** package calculates both Approximate and Sample Entropy much faster than package **pracma**. This is true not only for functions implemented in C (Figure 2), which were approximately 100 times faster, but even in R (Figure 3), which were about 20 times faster.

It can also be observed in Figure 4 and Figure 5 that the run-times of the new accelerated algorithms confirmed the assumption of the dependence of computation time on the analyzed time series disorder level. Interestingly, with these algorithms, the multiplicative difference between *sinTS* and *rnormTS* run-times for C functions was much greater (150 times) than for R functions (only 30 times) of the same **TSEntropies** package.

The computed values of all algorithms as well as their run-times were slightly lower for *powerTS* than for *rnormTS*, suggesting that there are some regularities in the power consumption waveform that distinguish it from a completely random signal. This proves that an eventual prediction of its development would make sense. Although this finding is not the purpose of this paper, it nevertheless indicates the possible use of this software.

Perhaps the most important result of testing this **TSEntropies** package seems to be that the computational times of the new Fast Approximate Entropy and Fast Sample Entropy algorithms are up to 500 times lower in the time series from the real world than the computational times of their original versions. This makes this software a really powerful tool for the fast searching for any regularities in huge amounts of data. Such type of search is needed in a wide range of fields. From the detection of epileptic seizures in the ECG, through the prediction of engine gear failure by mechanical vibration analysis, to such exotic issues as the search for intelligent life manifestations in radio signals from the surrounding universe. In all these cases (and many others), there is a need for rapid analysis of huge amounts of data. The new accelerated Fast Approximate Entropy and Fast Sample Entropy algorithms implemented in the **TSEntropies** package are perfect for this.

As a future development of this software, it seems appropriate to create a fully parallelized version, which would be intended mainly for processing big data on supercomputers. Another possible direction that this software development could take is to add a graphical output of the course of the calculated values along with the course of the analyzed time series, similar to those in Figure 1.

## Appendix A. Installation of TSEntropies Package

The **TSEntropies** package [9] is available from the Comprehensive R Archive Network (CRAN) at https://cran.r-project.org/package=TSEntropies. Therefore, the TSEntropies package can be installed in the standard way using the command:

R> install.packages("TSEntropies")

This package does not use any other R packages, so there is no need to worry about setting dependencies. However, the version of R itself should be at least 3.4.0 [11].

If the installation was successful, the package can be loaded by:

R> library(TSEntropies)

## Appendix B. TSEntropies Package Content and Implementation

The package contains functions for calculation of Approximate Entropy and Sample Entropy as well as their modified versions: Fast Approximate Entropy and Fast Sample Entropy. All these algorithms are implemented in two ways.

First, they are implemented as functions only in R. These functions include the suffix *_R* in their name. Then, these algorithms are implemented again in R, but, in this implementation, R serves only as a wrapper for hidden internal functions written in C. As it will be seen in the next chapter, using this way a significant acceleration of the calculation is achieved. Functions created in this way are indicated by an *_C* suffix.

The package also includes functions without any suffix in their name. These functions only trigger functions of the same name, but with the suffix *_C* and pass them the very same parameters. These alias functions are here to make it easier to use those faster versions of implementation. If someone wants to use functions written purely in R, then they must explicitly use functions with the suffix *_R*. However, in terms of speed, this option is not recommended.

The structure of the parameters is essentially the same for all implemented functions. The only difference is in the case of functions implementing modified algorithms in the default value of the last parameter *r*. This parameter determines the highest value of the distance between sub-sequences that are still considered as similar. For the original algorithms, it is recommended to set this parameter to a value corresponding to 0.2 times the standard deviation of the analyzed time series. However, in numerical experiments with modified algorithms, it has been found that it is more appropriate for them to set this parameter to a value equal to 0.15 times this standard deviation.

The remaining parameters, as well as their default values, are the same for all package functions. The analyzed time series, denoted as TS, is always the first and mandatory parameter. It should be in the form of some numeric R vector.

An important parameter is the dimension dim, which is optional. This parameter corresponds to the variable *m* in all the above formulas. As it can be seen from the package listing below, its default value is 2.

For clarity, here is a list of all the functions of this package and their syntax usage:

ApEn(TS, dim = 2, lag = 1, r = 0.2 * sd(TS))

ApEn_C(TS, dim = 2, lag = 1, r = 0.2 * sd(TS))

ApEn_R(TS, dim = 2, lag = 1, r = 0.2 * sd(TS))

SampEn(TS, dim = 2, lag = 1, r = 0.2 * sd(TS))

SampEn_C(TS, dim = 2, lag = 1, r = 0.2 * sd(TS))

SampEn_R(TS, dim = 2, lag = 1, r = 0.2 * sd(TS))

FastApEn(TS, dim = 2, lag = 1, r = 0.15 * sd(TS))

FastApEn_C(TS, dim = 2, lag = 1, r = 0.15 * sd(TS))

FastApEn_R(TS, dim = 2, lag = 1, r = 0.15 * sd(TS))

FastSampEn(TS, dim = 2, lag = 1, r = 0.15 * sd(TS))

FastSampEn_C(TS, dim = 2, lag = 1, r = 0.15 * sd(TS))

FastSampEn_R(TS, dim = 2, lag = 1, r = 0.15 * sd(TS))

All of these functions return a value of type *numeric*. Examples of use may be as follows.

A simple example of calculating the Approximate Entropy of a random time series with a normal probability distribution using a function implemented in C:

R> library(TSEntropies)

R> rnormTS <- rnorm(1000)

R> ApEn(rnormTS)

[1] 1.61967

R> ApEn(rnormTS, r = 0.1*sd(rnormTS))

[1] 1.07962

R> ApEn(rnormTS, dim = 4, r = 0.5*sd(rnormTS))

[1] 0.8241541

or an example of calculating the Fast Sample Entropy of a time series whose values are derived from the course of a sine wave using a function implemented in R:

R> library(TSEntropies)

R> sinTS <- sin(seq(0,100*pi,pi/10))

R> FastSampEn_R(sinTS)

[1] 0.003059666

R> FastSampEn_R(sinTS, r = 0.3*sd(sinTS))

[1] 0.003047234

R> FastSampEn_R(sinTS, dim = 5, r = 0.4*sd(sinTS))

[1] 0.001019888

It is similar for *ApEn_C()*, *ApEn_R()*, *SampEn()*, *SampEn_C()*, *SampEn_R()*, *FastApEn()*, *FastApEn_C()*, *FastApEn_R()*, *FastSampEn()*, and *FastSampEn_C()* functions.
